# Structural Flexibility in Activated Carbon Materials Prepared under Harsh Activation Conditions

**DOI:** 10.3390/ma12121988

**Published:** 2019-06-20

**Authors:** Fabiano Gomes Ferreira de Paula, Ignacio Campello-Gómez, Paulo Fernando Ribeiro Ortega, Francisco Rodríguez-Reinoso, Manuel Martínez-Escandell, Joaquín Silvestre-Albero

**Affiliations:** 1 Laboratorio de Materiales Avanzados, Departamento de Química Inorgánica-IUMA, Universidad de Alicante, Ctra. San Vicente-Alicante s/n, E-03690 San Vicente del Raspeig, Spain; fabianogfp@gmail.com (F.G.F.d.P.); nachocampeg@gmail.com (I.C.-G.); reinoso@ua.es (F.R.-R.); manolo.m@ua.es (M.M.-E.); 2 Universidade Federal de Minas Gerais (UFMG), Av. Antônio Carlos 6627, Belo Horizonte, Minas Gerais 31270-901, Brazil; 3 Departamento de Química, Centro Federal de Educaçao Tecnológica de Minas Gerais, Av. Amazonas 5253, Nova Suíça, Belo Horizonte 30421-169, Brazil; pauloortega@cefetmg.br

**Keywords:** activated carbon, structural flexibility, sensing selectivity, hydrocarbon and alcohol adsorption, electrical conductivity

## Abstract

Although traditionally high-surface area carbon materials have been considered as rigid structures with a disordered three dimensional (3D) network of graphite microdomains associated with a limited electrical conductivity (highly depending on the porous structure and surface chemistry), here we show *for the first time* that this is not the case for activated carbon materials prepared using harsh activation conditions (e.g., KOH activation). In these specific samples a clear structural re-orientation can be observed upon adsorption of different organic molecules, the structural changes giving rise to important changes in the electrical resistivity of the material. Whereas short chain hydrocarbons and their derivatives give rise to an increased resistivity, the contrary occurs for longer-chain hydrocarbons and/or alcohols. The high sensitivity of these high-surface area carbon materials towards these organic molecules opens the gate towards their application for sensing devices.

## 1. Introduction

Activated carbon materials have experienced a renovated interest in the last few years in fields such as electrochemical energy storage (supercapacitors and batteries), sensing devices, etc. [1,2,3,4,5,6]. The high interest of activated carbons for these applications is supported by their excellent textural properties (well-developed microporous structure and extremely large Brunauer-Emmet-Teller (BET) surface area), their tunable porosity and surface chemistry, low cost, their excellent mechanical properties to be conformed into electrodes, and their structural stability under different media (gas or liquid electrolyte), although their electrical conductivity is frequently very limited [7]. One of the basic criteria to identify the optimum carbon material for these applications (in terms of capacity, sensitivity, and selectivity) concerns the perfect knowledge of i) the porous structure of the synthesized carbon (presence of micropores and mesopores), ii) the surface chemistry (presence of functional groups on the carbon surface), and iii) the inner carbon structure (e.g., graphitization degree, size of the graphite microdomains, etc.). These characteristics will define among others the selective adsorption and electrochemical response (sensitivity) of the activated carbon devices. 

Contrary to zeolites and metal-organic framework materials where structural flexibility is widely accepted [8,9], activated carbon is assumed to keep a rigid un-changed structure under operando conditions, so that the structural parameters obtained from the characterization of the as-synthesized activated carbon (X-ray diffraction (XRD), gas adsorption at cryogenic temperatures, Raman spectrometry, etc.) can be used to understand their physico-chemical performance. Although this is true for many carbon materials, there are already some studies in the literature that anticipate a certain degree of structural deformation upon adsorption [10,11,12]. For instance, Kowalczyk et al. reported a 0.14 vol. % expansion upon CO_2_ adsorption at atmospheric pressure in a carbon xerogel. Despite the relevance of these structural changes taking place at the carbon skeleton upon an external stimulus and their potential effect in the macroscopic properties of the material, these changes, if any, have been commonly ignored. With this in mind, this manuscript aims to evaluate potential structural changes in activated carbon materials upon adsorption by combining XRD and Raman spectrometry. To this end, different carbon materials, coming from lignocellulosic or petroleum residues, and prepared under different chemical activation routes (low and high temperature, and different activating agents), will be compared. The effect of these structural changes in the final resistivity of the activated carbon material will be discussed. 

## 2. Experimental Section

Three different activated carbon precursors have been selected for this study: Two lignocellulosic-based materials (LAC: peach stones and cupuoaçu stones) and a petroleum residue (PAC). Lignocellulosic precursors have been activated using chemical activation with H_3_PO_4_ (LACx-A y) and KOH (LACx-K y), where “x” corresponds to peach (P) or cupuoaçu (C) and “y” identifies the activation temperature, while the petroleum residue-based carbon has been prepared by chemical activation with KOH (PAC-K y). 

Briefly, sample LACP-A 450 was prepared from peach stones using a chemical activation with H_3_PO_4_ (impregnation ratio x_p_ = 0.21 g P/g precursor). Activation was performed at 450 °C for 2 h under a nitrogen flow (80 mL/min) using a heating rate of 1 °C /min. Afterwards, the product was washed with distilled water until no reactant was present [13]. To identify the effect of the activation temperature and the activating agent in the same precursor, peach stones were also activated with H_3_PO_4_ at 800 °C, following the same recipe described above (LACP-A 800), and with KOH at 500 °C (LACP-K 500) and 800 °C (LACP-K 800). In the specific case of the KOH activated samples, the lignocellulosic residue was first carbonized at 500 °C for 2 h in a nitrogen atmosphere (100 mL/min). Afterwards, the sample was activated using KOH in a 3:1 ratio (KOH: precursor) at a final temperature of 500 °C or 800 °C for 2 h (nitrogen flow 100 mL/min). Finally, the samples were washed with 10 wt.% HCl and distilled water until neutral pH.

In order to evaluate the effect of the lignocellulosic residue, an additional sample LACC-K 700 was prepared from cupuoaçu stones from the Amazonia region. This sample was prepared following the same procedure described above and an activation temperature of 700 °C. 

To evaluate the effect of the carbon precursor, sample PAC-K 800 was prepared using mesophase pitch (VR-vacuum residue) obtained as a petroleum residue. The synthesis involved a pyrolysis step at 460 °C under 1 MPa of N_2_ for 1.5 h, followed by an activation treatment with KOH (3:1 wt.% activation ratio) at 800 °C for 2 h under a nitrogen flow (100 mL/min). The final material was washed with HCl (10 wt.%) and distilled water to completely remove the residual potassium [14].

The textural properties of the synthesized carbon materials (N_2_ isotherms at −196 °C) were measured using a home-made manometric equipment designed and constructed by the Laboratorio de Materiales Avanzados (LMA) group and now commercialized by Gas-to-Materials (http://www.g2mtech.com). Before the adsorption measurements, samples were treated in ultrahigh-vacuum at 250 °C for 4 h. 

Upon characterization of the textural properties, the different samples were impregnated using different organic molecules. Before the impregnation, samples were thermally treated at 80 °C for 24 h in an oven to remove humidity. The impregnation of the activated carbon was performed by immersing the carbon in an excess of the corresponding liquid molecule (n-nonane, n-hexane, hexanol, and octanol) at room temperature for 24 h (unless otherwise stated). Afterwards, the samples were filtered and dried at 60 °C under low vacuum for 1 h before being used for further characterization. The thermal treatment under these conditions is mandatory to remove the excess solvent from the external surface and meso-/macropores, thus leaving only the internal narrow microporosity filled with the organic molecule [15].

The crystallinity of the synthesized and impregnated activated carbons was evaluated using a Bruker D8-Advanced equipment using a X-ray KRISTALLOFLEX K 760–80 F generator with a copper anode. Raman spectroscopy studies were performed in a Jasco NRS-5100 spectrometer working with a laser of 532 nm and a CCD detector (resolution 6.83 cm^−1^). 

The electrical conductivity was measured in disc-shaped monoliths using a four-point probe system (Keithley Instruments, Model 238 High Current Source Measure Unit, USA). Before the conductivity measurements, the discs were prepared by mixing 90 wt.% of the activated carbon and 10 wt.% of polytetrafluoroethylene (≈ 30 mg of total mass for LACC-K and PAC-K and ≈ 70 mg for LAC-A) using a stainless steel mold of 1.32 cm^2^ and at a pressure of 454.5 × 10^3^ kPa. The calculation of resistivity and conductivity was done using correction factors taken from the literature [16].

## 3. Results and Discussion

### 3.1. Effect of the Carbon Precursor

The textural properties of the synthesized activated carbons were evaluated using nitrogen adsorption at cryogenic temperature. Supplementary Figure S1 shows the adsorption/desorption isotherms at −196 °C for some selected samples, i.e., LACP-A 450, LACC-K 700, and PAC-K 800 samples. The selection of these samples prepared from a different precursor (lignocellulosic and petroleum), and using a different activation procedure (phosphoric acid vs. KOH) will allow a pre-evaluation of the structural flexibility in activated carbon materials. As expected, KOH-activated carbons exhibit a larger development of microporosity and a larger associated BET surface area compared to the conventional H_3_PO_4_ activated sample. Textural parameters for the three samples are collected in Table 1. As it can be observed, the samples are preferentially microporous (type I isotherm; V_0_ ≈ V_T_), BET surface area ranging from 1200 m^2^/g, for the lignocellulosic-based H_3_PO_4_ sample, up to 2500 m^2^/g, for the lignocellulosic-based KOH activated sample.

**Figure 1:**
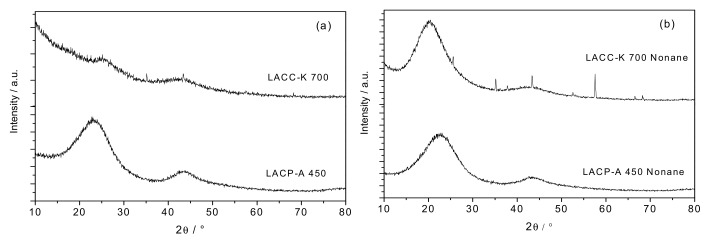
X-ray diffraction (XRD) profiles for the lignocellulosic-based activated carbons (**a**) before and (**b**) after adsorption of n-nonane.

The crystallographic structure of the synthesized activated carbon materials has been evaluated using X-ray diffraction (XRD) measurements. Figure 1a shows the XRD pattern for the lignocellulosic activated carbons, LACP-A 450, and LACC-K 700. As it can be appreciated, whereas the sample activated with the conventional H_3_PO_4_ treatment preserves the dominant feature of graphite at 2θ = 23° and 43°, attributed to the (002) and (10) diffractions, the KOH activated samples exhibits a rather flat profile (only two tiny shoulders can be appreciated at 25° and 43°). The fact that the XRD pattern upon KOH activation is almost completely washed-out is in close agreement with previous studies by Takahata et al., preferentially for high-surface area KOH activated samples [17]. The shift of the (002) peak to lower angles in sample LACP-A 450 compared to pure graphite (23° vs. 26°) clearly anticipates the defective nature of the synthesized carbon, i.e., the presence of small graphite microdomains with a short range order for the graphene layers [18]. However, the complete disruption of the crystallographic ordering in the KOH activated sample denotes, in addition, the aggressive nature of the activation treatment, in close agreement with the proposed activation mechanism [19,20,21]. According to the studies described in the literature, the activation process with KOH involves a series of exothermic reactions (sometimes explosive reactions) via the intercalation of metallic potassium in between the graphene layers and the evolution of hydrogen, thus given rise to the complete exfoliation of the final carbon material. Equation (1) shows one of the main reactions taken place in the activation process. However, a detailed analysis of the KOH activation process can be found elsewhere [19,20,21].

(1) $$6\mathrm{KOH}+C↔2K+3H_{2}+2K_{2}{\mathrm{CO}}_{3}$$

On the contrary, the activation treatment with H_3_PO_4_ implies less aggressive processes, mainly depolymerization of cellulose catalyzed by phosphoric acid, followed by dehydration and condensation, leading to more aromatic and reactive products, with some cross-linking [13]. 

Surprisingly, the structural disordering and the associated flat XRD pattern drastically change upon adsorption of a linear hydrocarbon, such as n-nonane. As it can be appreciated in Figure 1b, whereas the XRD profile for the LACP-A 450 sample does not exhibit any significant modification after incorporation of n-nonane (the broad contributions at 23° and 43° are fully preserved), the XRD profile for the KOH-activated lignocellulosic precursor drastically changes, with new broad contributions appearing at 2θ = 20° and 43°. The development of these new contributions is more evident for the peak at 20°. This observation anticipates an important structural re-structuring of the graphite/graphene microdomains upon adsorption of the hydrocarbon. Furthermore, the *d*-spacing for the (002) plane shifts from 0.386 nm in the LACP-A 450 sample to 0.443 nm in the LACC-K 700 sample (the interlayer spacing *d*_002_ for highly oriented graphite is 0.335 nm). These results suggest that the re-structured material after KOH activation is highly defective, compared to the conventional lignocellulosic-based activated carbon. However, the increased *d*_002_ spacing due to n-nonane intercalation within the graphene layers cannot be ruled out. In fact, the value of 0.443 nm is highly above previous values described in the literature for graphitizable and non-graphitizable materials [22]. Last but not least, the XRD profile for LACC-K 700 also shows the appearance of some sharp contributions at 25°, 35.1°, 43.4°, and 57.4°. Since these contributions are also present in the dry material, they cannot be attributed to n-nonane. Although the ash content of the synthesized activated carbon after acid washing is less than 0.01%, we cannot exclude these peaks (corresponding to Al_2_O_3_) to come from the mineral content from the raw cupuaçu stones.

Similar experiments were performed in the petroleum-pitch derived activated carbon prepared through a chemical activation process with KOH (sample PAC-K 800). As it can be observed in Supplementary Figure S2, the original raw material exhibits a rather flat profile, in close agreement with sample LACC-K 700. Once again, the adsorption of n-nonane gives rise to a sudden change in the XRD pattern with new broad contributions at 2θ = 20° and 43°, similar to the ones observed for sample LACC-K 700. Interestingly, the results are perfectly reproducible after 1 h or 24 h of contact time between the activated carbon skeleton and the hydrocarbon, i.e., the inner re-structuring process seems to be a sudden response of the material upon adsorption/impregnation. In other words, the response factor of the internal carbon structure upon an external stimulus (upon adsorption) must be a fast process, thus opening the gate towards the application of this phenomenon in sensing devices. A closer look to Supplementary Figure S2 confirms that 1 h is enough to fully develop the broad (002) contribution, the peak position being rather similar after 1 h and 24 h.

**Figure 2:**
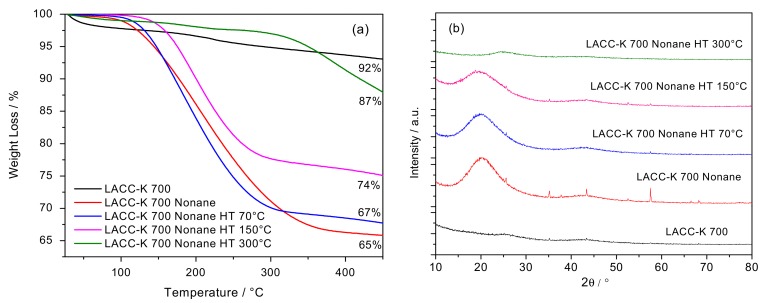
(**a**) Thermogravimetric analysis of sample LACC-K 700 before and after exposure to n-nonane, and LACC-K 700 sample after different thermal treatments under inert atmosphere; (**b**) XRD pattern for the samples evaluated.

To further ascertain the role of the hydrocarbon in the structural re-ordering, sample LACC-K 700 pre-adsorbed with n-nonane has been submitted to different thermal treatments at ranging temperatures to selectively remove the hydrocarbon from the inner microporous structure. The thermogravimetric (TG) analyses have been complemented by XRD of the thermally treated samples. Thermogravimetric analysis reported in Figure 2 show that the raw material is able to adsorb ca. 35 wt.% of hydrocarbon, in close agreement with the highly developed microporous structure (characteristic of KOH activated samples). Assuming the density of n-nonane (0.72 g/cm^3^), an adsorption capacity of 35 wt.% denotes that the micropores in sample LACC-K 700 are filled with n-nonane. Compared to the dried activated carbon, the incorporation of n-nonane gives rise to the broad contributions at 20° and 43°, as described above. A thermal treatment at 70 °C for 24 h under ultrahigh-vacuum conditions does not modify the amount of n-nonane adsorbed in the micropores of the activated carbon (onset desorption temperature is ca. 120 °C), neither the XRD pattern [15]. Expectedly, a further increase in the outgassing temperature to 150 °C for 24 h gives rise to partial loss of pre-adsorbed n-nonane (ca. 26 wt.% n-nonane remains in the structure), the remaining n-nonane occluded in the micropores having a lower effect in the XRD pattern, i.e., a decrease in the intensity and a widening of the (002) contribution at 20° can be clearly appreciated. Last but not least, the complete removal of n-nonane at 300 °C completely washes-out the XRD pattern with no appreciable contributions over the whole 2θ range evaluated. These results anticipate the crucial role of the adsorbed hydrocarbon in the observed structural changes.

In summary, these results clearly anticipate that chemical activation with KOH of different carbon precursors (for instance, a lignocellulosic or a petroleum residue) constitutes an aggressive activation route with a complete disruption of the 3D ordering and crystallinity in the final material. However, despite this a priori harsh effect, the long-range structural ordering can be easily and reversible recovered upon the incorporation of a hydrocarbon in the inner microstructure. 

**Figure 3:**
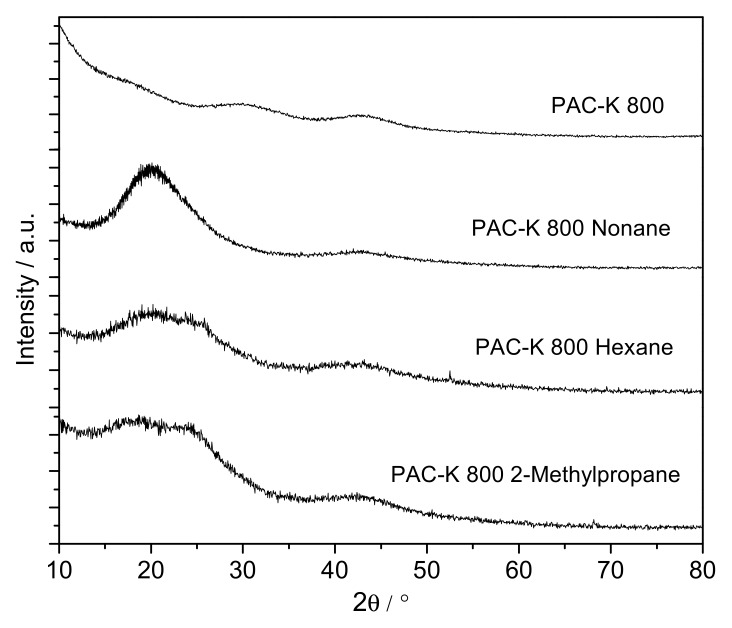
XRD profiles for the petroleum-pitch activated carbon (PAC-K 800) before and after incorporation of different hydrocarbons.

An open question at this point concerns the effect that the nature of the adsorbed molecule can have in the final structural re-structuring. To this end, the crystallographic pattern of sample PAC-K 800 has been evaluated as prepared and after incorporation of different linear/branched hydrocarbons (n-nonane, n-hexane and 2-methyl-propane) after 24 h. XRD patterns reported in Figure 3 clearly show that the long-range re-structuring of the activated carbon microstructure highly depends on the nature of the hydrocarbon pre-adsorbed. Interestingly, these results provide some evidences about the selective nature of this phenomenon. Whereas n-nonane provides a well-defined symmetric contribution at 20°, shorter chain hydrocarbons (e.g., n-hexane and 2-methylpropane) do not. For these two hydrocarbons, a wide contribution develops upon adsorption with smooth shoulders at 2θ ≅ 18° and 23°, thus reflecting the absence of a long-range high quality ordering for shorter range hydrocarbons. Although these results suggest a certain re-structuring upon adsorption of short-chain hydrocarbons, the presence of two shoulders could be associated with the development of two differently oriented microdomains. 

To confirm that the adsorbed hydrocarbons remain in the microstructure after the drying step, TG curves for LACC-K 700 and PAC-K 800 samples after impregnation with n-nonane and hexane, and vacuum drying have been performed (Supplementary Figure S3). As it can be observed, the weight loss is rather similar for both hydrocarbons in both materials, thus confirming that the hydrocarbons remain in the micropores. Last but not least, Supplementary Figure S4 shows that the re-structuring order produced by n-nonane can be reversibly removed after washing the pre-impregnated material with H_2_O or even more efficiently with acetone (washing was performed at 25 °C per triplicate using 50 mL of pure solvent). 

### 3.2. Effect of The Activation Temperature

**Figure 4:**
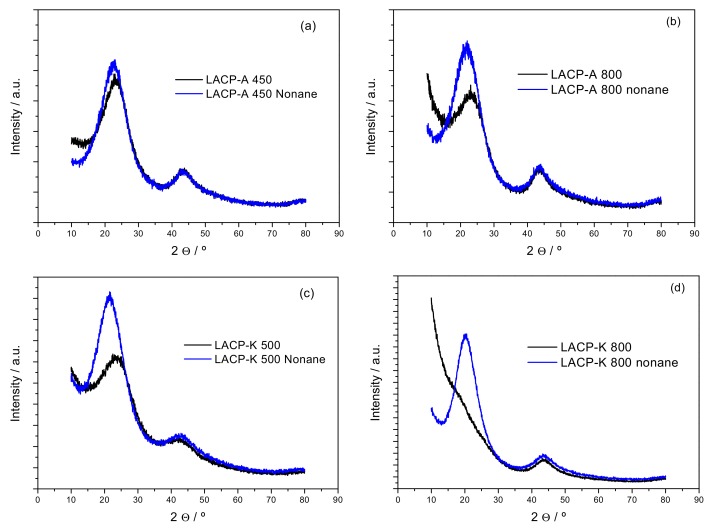
XRD profiles for the peach stone-based activated carbon after (**a**, **b**) acid (LACP-A) and (**c**, **d**) KOH (LACP-K) activation prepared at (**a**, **c**) low and (**b**, **d**) high activation temperature, and before and after incorporation of different hydrocarbons.

In order to evaluate the effect that the activation temperature has in these structural phenomena, the XRD patterns for samples prepared from peach stones (H_3_PO_4_ activated: LACP-A 450, LACP-A 800; KOH activated: LACP-K 500 and LACP-K 800) have been compared before and after the pre-impregnation with n-nonane (Figure 4). In addition, the textural characteristics of the synthesized samples can be compared in Supplementary Figure S5. As expected, samples activated at high temperature (800 °C), either using acid or KOH activation, exhibit a higher development of porosity, and a larger BET surface area. XRD data of the as-synthesized carbon materials confirms previous findings, i.e., samples activated with H_3_PO_4_ preserve the two contributions at 23° and 43°, whereas this is not the case for KOH activated samples, especially after the activation treatment at high temperature. In any case, pre-impregnation with n-nonane gives rise to a sudden development of the 2 theta contribution at 20°–21°, this observation being more drastic in sample LACP-K 800. However, this structural re-orientation is not only evident in the activated carbons prepared by KOH activation at low and high temperature but, in a certain way, in the high temperature acid treated sample. 

Consequently, these results confirm that the structural flexibility appreciated in activated carbon material upon hydrocarbon adsorption is an intrinsic property of the structure itself, independently of the nature of the carbon precursor and the activation treatment used. However, these changes are more easily appreciated in activated carbons with a random orientation of the graphite microdomains, i.e., samples submitted to a harsh activation treatment. This is the case, for instance, in samples activated with KOH at low and high temperatures while, in the specific case of H_3_PO_4_ activated carbons, only in samples obtained at high temperature is appreciated. 

### 3.3. Raman Spectrometry for Samples LACC-K 700 and PAC-K 700 

**Figure 5:**
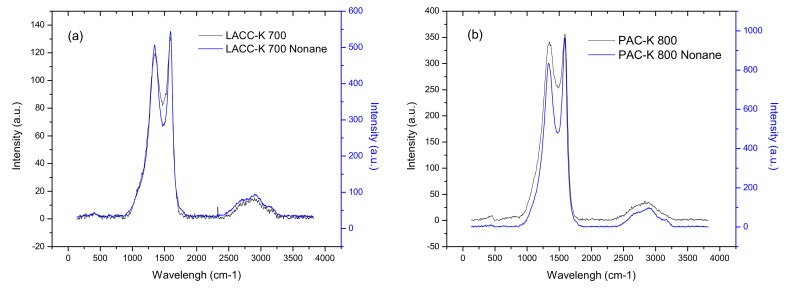
Raman spectra for the KOH activated carbons ((**a**) LACC-K 700 and (**b**) PAC-K 800) before and after incorporation of n-nonane.

Another potential technique to evaluate fine structural changes in activated carbons is Raman spectrometry. In the specific case of polycrystalline graphite, Raman spectra exhibits two sharp rotational contributions, the so-called G-band around 1580 cm^−1^ and D-band (disorder band) around 1355 cm^−1^, ascribed to the E_2g_ and A_1g_ in-plane rotational modes, respectively. Interestingly, the position and width of these bands depends on the material carbonization degree and the degree of disorder (porosity, crystallite size distribution, etc.). Furthermore, the intensity of the D band inversely scales with the size of the microcrystals, i.e., the degree of long-range ordering, so that the intensity ratio I_D_/I_G_ can be correlated with the reciprocal of the crystallite size along basal plane measured from XRD. Figure 5 shows the Raman spectra for two KOH-activated carbons (LACC-K 700 and PAC-K 800) before and after incorporation of n-nonane. In both cases, the spectra exhibit two sharp contributions at 1344 cm^−1^ and 1591 cm^−1^, attributed to the D and G-bands, respectively. In addition, these spectra show a broad shoulder in the 2600–3400 cm^−1^ range, due to the G and D bands overtones and the D-G inter-combination band [23].

As it can be appreciated, the incorporation of n-nonane does not have any effect in the position of the D and G bands for both activated carbons. However, important differences can be appreciated in the relative intensities, preferentially for sample PAC-K 800 (an important decrease in the D-band intensity at 1344 cm^−1^ can be clearly appreciated). Indeed, the I_D_/I_G_ band ratio is rather similar for the LACC-K 700 sample after n-nonane incorporation (0.93 vs. 0.91), while large differences are observed for PAC-K 800 sample (0.86 vs. 0.96). The larger I_D_/I_G_ ratio after n-nonane adsorption in sample PAC-K 800 clearly confirms an increased long-range ordering upon adsorption, in close agreement with XRD data.

### 3.4. Electrical Conductivity Measurements

**Figure 6:**
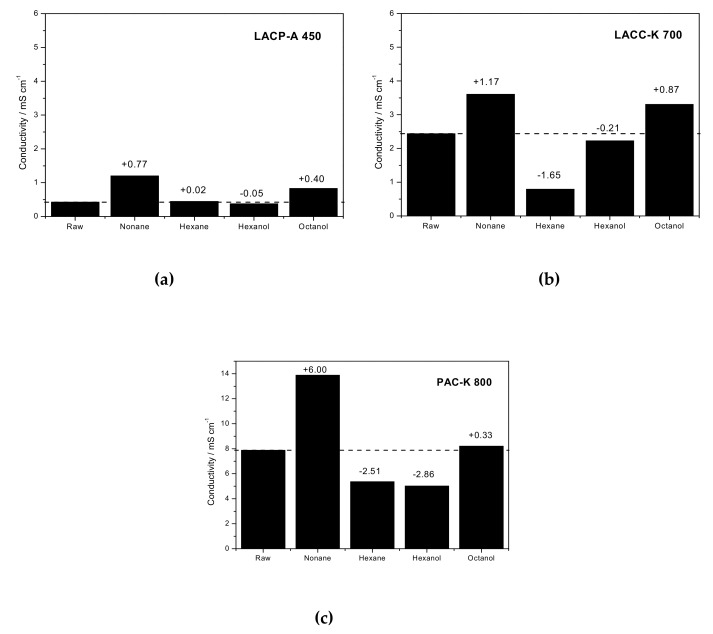
Electrical conductivity (mS/cm) measured for the three activated carbons: **(a)** LACP-A 450; **(b)** LACC-K 700 and **(c)** PAC-K 800, evaluated before (raw) and after adsorption of different hydrocarbons or alcohols (nonane, hexane, hexanol and octanol).

Experimental results described above have shown that activated carbon materials prepared under harsh activation conditions (e.g., KOH activation) can exhibit intrinsic structural re-structuring upon hydrocarbon adsorption, preferentially for long chain molecules. If an internal re-structuring is the main reason to explain the aforementioned observations, it is easy to understand that these structural changes must be reflected in important changes in the macroscopic properties of the evaluated activated carbon materials. Among the different potential properties susceptible to be affected by these order/disorder changes, one of the most sensitive to the long-range order of the electronically coherent domains, and extremely important from a technological point of view, will be the electrical resistivity. Although activated carbons are considered materials with a high resistivity due to the intrinsic stochastic ordering of the graphite microdomains, the above described changes could have an important effect in these properties. To evaluate this point, Figure 6 compares the electrical conductivity (inverse of resistivity) for some of the activated carbon materials developed in this study, in monolith-shape (see experimental section and Supplementary Table S1 for further details) before and after adsorption of different organic molecules. The initial conductivity (or resistivity) of the synthesized activated carbons is in close agreement with their internal structure, i.e., lignocellulosic materials exhibit a higher resistivity compared to a graphitizable petroleum-pitch derived carbon. The existence of extended graphite-based microdomains in the petroleum residue favors the electrical conductivity. Indeed, sample LACP-A 450 exhibits the lowest conductivity (resistivity as high as 2.34 kΩ·cm), followed by sample LACC-K 700 with a resistivity of 410 Ω·cm and sample PAC-K 800 with a resistivity around 127 Ω·cm (see resistivity measurements in Supplementary Figures S6–S10). These resistivity values are very similar to those described in the literature by Adinaveen et al. for lignocellulosic-based carbon materials [24]. Interestingly, the incorporation of an organic molecule in the microporous structure of these samples gives rise to important changes in the conductivity, these changes being highly sensitive to the nature of the adsorbed molecule (including the chain length). Although these changes are small for the LACP-A 450 sample, they already anticipate a clear tendency, i.e., longer chain hydrocarbons and/or alcohols highly improve the conductivity, while shorter hydrocarbons and/or alcohols give rise to a decreased conductivity. This tendency can be more clearly appreciated for sample LACC-K 700, and even more for PAC-K 800. In both cases, n-nonane and octanol give rise to an improved conductivity while n-hexane and hexanol increases the resistivity. These effects are extremely large for PAC-K 800 sample with an increase of ≈ 6 mS/cm after incorporation of n-nonane in the micropores. The larger conductivity changes in sample LACC-K 700 and, even more important, for PAC-K 800 sample are in close agreement with the structural changes anticipated by XRD and Raman. 

Previous studies described in the literature have shown that the mechanism of electronic conduction in activated carbon materials does not resemble neither that of metals nor of semiconductors [24,25]. It is rather a “hopping” mechanism between coherent domains, so that the final conductivity highly depends on the long-range ordering or graphitization degree [25]. More specifically, the turbostratic carbon structure is constituted by graphite-like domains containing one positive (p-) or negative (n-) excess charge, although they can also be uncharged. In this microdomains excess charge is delocalized and moves freely. However, the low conductivity in activated carbon materials is rather due to the restricted mobility in between twisted domains. The weak electronic overlap at the interface force charges to travel by tunneling, through a “hopping” mechanism [26,27]. At this point it is important to highlight that the modification of the carbon structure through the incorporation of functional groups can be used to modify the electron and/or hole mobility within the graphite microdomains and, indirectly, the electrical resistivity [5]. Depending on the nature of the functionality, i.e., electron-donating or electron-deficient, the resistivity would increase or decrease accordingly. Furthermore, it is also true that the adsorption of organic molecules (for instance, NH_3_ with a lone pair electron) in the surface of carbon materials can induce changes in the materials´ resistivity through electron transfer processes (for instance, the electron donating properties of ammonia). Despite these premises, the combination of XRD and Raman with resistivity measurements upon n-nonane adsorption, taking into account that hydrocarbons are not prone to induce important electronic changes in the carbon structure, clearly show *for the first time* that activated carbon materials are not as rigid as expected, the internal re-structuring upon gas/liquid adsorption playing a crucial role in the final electrical performance of the material. Indeed, the large improvement in the conductivity of sample PAC-K 800 (up to 6 mS/cm) can only be understood through the observed structural flexibility changes, anticipated by XRD and Raman measurements. On the other hand, the restricted conductivity upon adsorption of short-chain hydrocarbons and/or alcohols could be due to the presence of two differently oriented microdomains, thus limiting the “hopping” mechanism compared to the original carbon with a stochastic ordering (with higher probability for electron mobility in between graphite microdomains), in close agreement with XRD measurements.

Last but not least, it is important to highlight that the large selectivity to the hydrocarbon chain length and the nature of the organic molecule (hydrocarbon or alcohol) observed in Figure 6 opens the gate towards the application of KOH-activated carbon materials as sensing devices for the detection of organic molecules. 

## 4. Conclusions

Combination of XRD and Raman spectroscopy upon adsorption of different organic molecules shows that the inner structure of activated carbon materials prepared under harsh activation conditions (for instance, KOH activation) is not as rigid as expected. According to these results, the graphite microdomains that constitute the skeleton go through an internal reorientation upon adsorption of long-chain hydrocarbons/alcohols (e.g., n-nonane), associated with an important increase in the electrical conductivity. On the contrary, the adsorption of short-chain hydrocarbons/alcohols gives rise to a decrease in the conductivity compared to the raw material, in close agreement with the presence of two different microdomains as observed using XRD measurements. The high selectivity of this structural reorientation to the nature of the adsorbed molecule opens the gate towards the application of these carbons, preferentially KOH activated carbons from petroleum residue, in sensing devices.

## Figures and Tables

**Table 1:** Textural properties estimated from the N_2_ adsorption data at −196 °C. Total pore volume (V_T_) was estimated at p/p_0_ ≈ 0.95 and the micropore volume (V_0_) was estimated from the Dubinin–Radushkevich (DR) equation.

Sample	BET (m^2^/g)	V_T_(cm^3^/g)	V_0_(cm^3^/g)
LACP-A 450	1180	0.50	0.43
LACC-K 700	2490	1.01	0.94
PAC-K 800	2000	0.87	0.76

## Supplementary Materials

The following are available online at https://www.mdpi.com/1996-1944/12/12/1988/s1, Table S1: Resistance and R^2^ values obtained after fit linear of the voltage versus current curves in ohmic regime. For the calculation of resistivity from the resistance data, we used 0.9974 and 4.2753 as correction factors for thickness and diameter, respectively, according to the cited reference (Quim Nova 2002;25:639-47). Figure S1: N_2_ adsorption/desorption isotherms at 77 K for the three activated carbons evaluated. Figure S2: XRD pattern of the petroleum-based activated carbon (PAC-K 800) before and after n-nonane adsorption for 1 and 24 h. Figure S3: Thermogravimetric analysis (TG) of the LACC-K 700 and PAC-K 800 samples after pre-impregnation with nonane and hexane and a subsequent drying step at 60 °C for 1 h under low vacuum. Figure S4: XRD pattern of the petroleum-based activated carbon (PAC-K 800) before and after n-nonane adsorption and after a washing treatment with H_2_O and acetone (C_3_H_6_O). Figure S5: N_2_ adsorption/desorption isotherms at 77 K for the peach stones derived activated carbons evaluated (LACP-A and LACP-K) prepared at low and high activation temperatures. Figure S6: DC electrical resistivity measurements for PAC-K 800 sample before and after n-nonane adsorption using fourpoint probe method. The resistance values were obtained from the slope of the voltage versus current curves in ohmic regime. Figure S7: DC electrical resistivity measurements for PAC-K 800 sample after adsorption of hexane, hexanol and octanol using fourpoint probe method. The resistance values were obtained from the slope of the voltage versus current curves in ohmic regime. Figure S8: DC electrical resistivity measurements for LACC-K 700 sample before and after n-nonane adsorption using fourpoint probe method. The resistance values were obtained from the slope of the voltage versus current curves in ohmic regime. Figure S9: DC electrical resistivity measurements for LACC-K 700 sample after adsorption of hexane, hexanol and octanol using fourpoint probe method. The resistance values were obtained from the slope of the voltage versus current curves in ohmic regime. Figure S10: DC electrical resistivity measurements for LACP-A 450 before and after adsorption of n-nonane, hexane, hexanol and octanol using fourpoint probe method. The resistance values were obtained from the slope of the voltage versus current curves in ohmic regime.

## References

[1] Tai Z., Zhang Q., Liu Y., Liu H., Dou S. (2017). Activated carbon from the graphite with increased rate capability for the potassium ion battery. Carbon.

[2] Moreno N., Caballero A., Hernan L., Morales J. (2014). Lithium-sulfur batteries with activated carbons derived from olive stones. Carbon.

[3] Chmiola J., Yushin G., Dash R., Gogotsi Y. (2006). Effect of pore size and surface area of carbide derived carbons on specific capacitance. J. Power Sour..

[4] Hu C., Kirk C., Cai Q., Cuadrado-Collados C., Silvestre-Albero J., Rodriguez-Reinoso F., Biggs M.J. (2017). A high-volumetric-capacity cathode based on interconnected close-packed N-doped porous nanospheres for long-life lithium-sulfur batteries. Adv. Energy Mater..

[5] Travlou N.A., Seredych M., Rodriguez-Castellon E., Bandosz T.J. (2015). Activated carbon-based gas sensors: Effect of surface features on sensing mechanism. J. Mater. Chem. A.

[6] Arroyo-Gómez J.J., Villaroel-Rocha D., de Freitas-Araujo K.C., Martinez-Huitle C.A., Sapag K. (2018). Applicability of activated carbon obtained from peach stones as an electrochemical sensor for detecting caffeine. J. Electr. Chem..

[7] Rodriguez-Reinoso F., Marsh H. (2006). Activated Carbon.

[8] Bereciartua P.J., Cantín A., Corma A., Jordá J.L., Palomino M., Rey F., Valencia S., Corcoran E.W., Kortunov P., Ravikovitch P.I. (2017). Control of zeolite framework flexibility and pore topology for separation of ethane and ethylene. Science.

[9] Cuadrado-Collados C., Fernandez-Catala J., Fauth F., Cheng Y.Q., Daemen L.L., Ramirez-Cuesta A.J., Silvestre-Albero J. (2017). Understanding the breathing phenomena in nano-ZIF-7 upon gas adsorption. J. Mater. Chem. A.

[10] Kowalczyk P., Balzer C., Reichenauer G., Terzyk A.P., Gauden P.A., Neimark A.V. (2016). Using in-situ adsorption dilatometry for assessment of micropore size distribution in monolithic carbons. Carbon.

[11] Balzer C., Braxmeier S., Neimark A.V., Reichenauer G. (2015). Deformation of microporous carbon during adsorption of nitrogen, argon, carbon dioxide, and water studies by in situ dilatometry. Langmuir.

[12] Barreda D., Pérez-Mas A.M., Silvestre-Albero A., Casco M.E., Rudic S., Herdes C., Müller E.A., Blanco C., Santamaria S., Silvestre-Albero J. (2017). Unusual flexibility of mesophase pitch-derived carbon materials: An approach to the synthesis of graphene. Carbon.

[13] Molina-Sabio M., Rodríguez-Reinoso F. (2004). Role of chemical activation in the development of carbon porosity. Coll. Surf. A.

[14] Casco M.E., Martínez-Escandell M., Silvestre-Albero J., Rodríguez-Reinoso F. (2014). Effect of the porous structure in carbon materials for CO_2_ capture at atmospheric and high-pressure. Carbon.

[15] Oschatz M., Borchardt L., Rico-Francés S., Rodríguez-Reinoso F., Kaskel S., Silvestre-Albero J. (2013). Textural characterization of micro- and mesoporous carbons using combined gas adsorption and n-nonane preadsorption. Langmuir.

[16] Girotto E.M., Santos I.A. (2002). Medidas de resistividade elétrica DC em sólidos: Como efetuá-las correctamente. Quim. Nova.

[17] Takahata T., Toda I., Ono H., Ohshio S., Akasaka H., Himeno S., Kokubu T., Saitoh H. (2009). Detailed structural analyses of KOH activated carbon from waste coffee beans. Jpn. J. Appl. Phys..

[18] Manna K., Kumar Srivastava S., Mittal V. (2016). Role of enhanced hydrogen bonding of selectively reduced graphite oxide in fabrication of poly (vinyl alcohol) nanocomposites in water as EMI shielding materials. J. Phys. Chem. C.

[19] Lillo-Ródenas M.A., Cazorla-Amorós D., Linares-Solano A. (2003). Understanding chemical reactions between carbons and NaOH and KOH: An insight into the chemical activation mechanism. Carbon.

[20] Krol M., Gryglewicz G., Machnikowski J. (2011). KOH activation of pitch-derived carbonaceous materials-Effect of carbonization degree. Fuel Process. Technol..

[21] Martínez-Escandell M., Monteiro de Castro M., Molina-Sabio M., Rodriguez-Reinoso F. (2013). KOH activation of carbon materials obtained from the pyrolisis of ethylene tar at different temperatures. Fuel Process. Technol..

[22] Zhao J., Yang L., Li F., Yu R., Jin C. (2009). Structural evolution in the graphitization process of activated carbon by high-pressure sintering. Carbon.

[23] Bhoyate S., Ranaweera C.K., Zhang C., Morey T., Hyatt M., Kahol P.W., Ghimire M., Mishra S.R., Gupta R.K. (2017). Eco-friendly and high performance supercapacitors for elevated temperature applications using recycled tea leaves. Glob. Chall..

[24] Adinaveen T., Vijaya J.J., John Kennedy L. (2016). Comparative study of electrical conductivity of activated carbons prepared from various cellulose materials. Arab. J. Sci. Eng..

[25] Kastening B., Hahn M., Rabanus B., Heins M., zum Felde U. (1997). Electronic properties and double layer of activated carbon. Electrochim. Acta.

[26] Kastening B. (1998). A model of the electronic properties of activated carbon. Ber. Bunsenges. Phys. Chem..

[27] Sheng P., Klafter J. (1982). Hopping conductivity in granular disordered systems. Phys. Rev. B.

