# Supplementary material for: Structural Flexibility in Activated Carbon Materials Prepared under Harsh Activation Conditions
Source: Materials (Basel). 2019 Jun 20;12(12):1988. doi: 10.3390/ma12121988 (PMC6632014; doi:10.3390/ma12121988)
Supplement: Supplementary file 1 [file materials-12-01988-s001.pdf]

materials

Structural Flexibility in Activated Carbon Materials Prepared under Harsh Activation Conditions

Fabiano Gomes Ferreira de Paula[a,b], Ignacio Campello-Gómez[a], Paulo F.R. Ortega,[c] Francisco Rodríguez-Reinoso[a], Manuel Martínez-Escandell[a] and Joaquín Silvestre-Albero[a,*]

SUPPORTING INFORMATION

Table of content

Table S1. Resistance and R^2 values obtained after fit linear of the voltage versus current curves in ohmic regime. For the calculation of resistivity from the resistance data, we used 0.9974 and 4.2753 as correction factors for thickness and diameter, respectively, according to the cited reference (Quim Nova 2002;25:639-47)................*Page 3*

Figure S1. N_2 adsorption/desorption isotherms at 77 K for the three activated carbons evaluated................*Page 4*

Figure S2. XRD pattern of the petroleum-based activated carbon (PAC-K 800) before and after n-nonane adsorption for 1 and 24 hours................*Page 5*

Figure S3. Thermogravimetric analysis (TG) of the LACC-K 700 and PAC-K 800 samples after pre-impregnation with nonane and hexane and a subsequent drying step at 60ºC for 1h under low vacuum……………*Page 6*

Figure S4. XRD pattern of the petroleum-based activated carbon (PAC-K 800) before and after n-nonane adsorption and after a washing treatment with H_2O and acetone (C_3H_6O)................*Page 7*

Figure S5. N_2 adsorption/desorption isotherms at 77 K for the peach stones derived activated carbons evaluated................*Page 8*

Figure S6. DC electrical resistivity measurements for PAC-K 800 sample before and after n-nonane adsorption using fourpoint probe method. The resistance values were obtained from the slope of the voltage versus current curves in ohmic regime................*Page 9*

Figure S7. DC electrical resistivity measurements for PAC-K 800 sample after adsorption of hexane, hexanol and octanol using fourpoint probe method. The resistance values were obtained from the slope of the voltage versus current curves in ohmic regime................*Page 10*

Figure S8. DC electrical resistivity measurements for LACC-K 700 sample before and after n-nonane adsorption using fourpoint probe method. The resistance values were obtained from the slope of the voltage versus current curves in ohmic regime…................*Page 11*

Figure S9. DC electrical resistivity measurements for LACC-K 700 sample after adsorption of hexane, hexanol and octanol using fourpoint probe method. The resistance values were obtained from the slope of the voltage versus current curves in ohmic regime................*Page 12*

Figure S10. DC electrical resistivity measurements for LACP-A 450 before and after adsorption of n-nonane, hexane, hexanol and octanol using fourpoint probe method. The resistance values were obtained from the slope of the voltage versus current curves in ohmic regime……..*Page 13*

Table S1. Resistance and R^2 values obtained after fit linear of the voltage versus current curves in ohmic regime. For the calculation of resistivity from the resistance data, we used 0.9974 and 4.2753 as correction factors for thickness and diameter, respectively, according to the cited reference (Quim Nova 2002;25:639-47).

Sample:	Resistance / Ω:	R^2:	Resistivity / Ω cm:
PAC-K 800 Raw	1070	0,9996	126,89
PAC-K 800 Nonane	880	0,9993	72,05
PAC-K 800 Hexane	2090	0,9997	186,26
PAC-K 800 Hexanol	1720	0,9836	199,50
PAC-K 800 Octanol	1360	0,9988	121,79
LACC-K 700 Raw	4294	1	410,15
LACC-K 700 Nonane	2792	1	277,40
LACC-K 700 Hexane	4980	1	1265,64
LACC-K 700 Hexanol	2590	0,9996	449,50
LACC-K 700 Octanol	3300	1	302,54
LACP-A 450 Raw	40600	0,9999	2337,20
LACP-A 450 Nonane	9560	0,9964	831,62
LACP-A 450 Hexane	11670	1	2244,31
LACP-A 450 Hexanol	6530	0,9999	2645,29
LACP-A 450 Octanol	5150	0,9996	1203,44

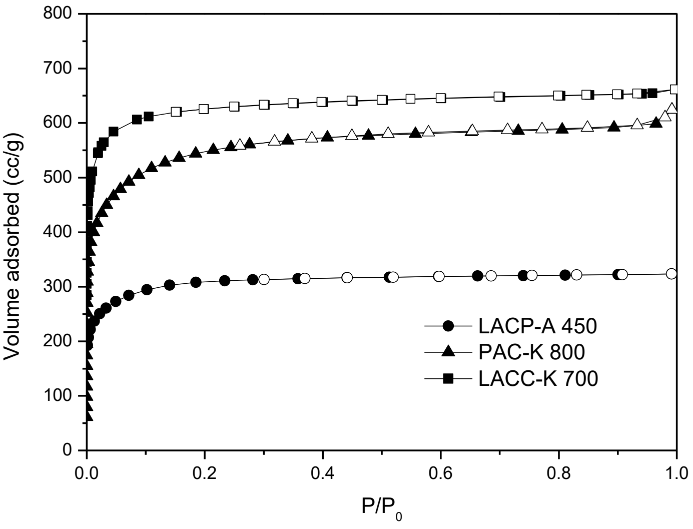

Figure S1. N_2 adsorption/desorption isotherms at 77 K for the three activated carbons evaluated.

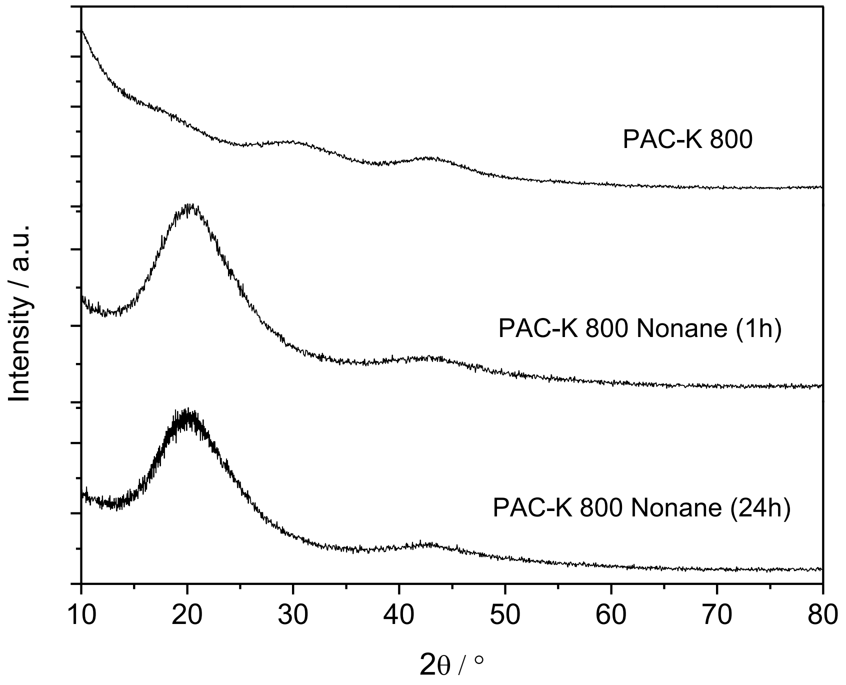

Figure S2. XRD pattern of the petroleum-based activated carbon (PAC-K 800) before and after n-nonane adsorption for 1 and 24 hours.

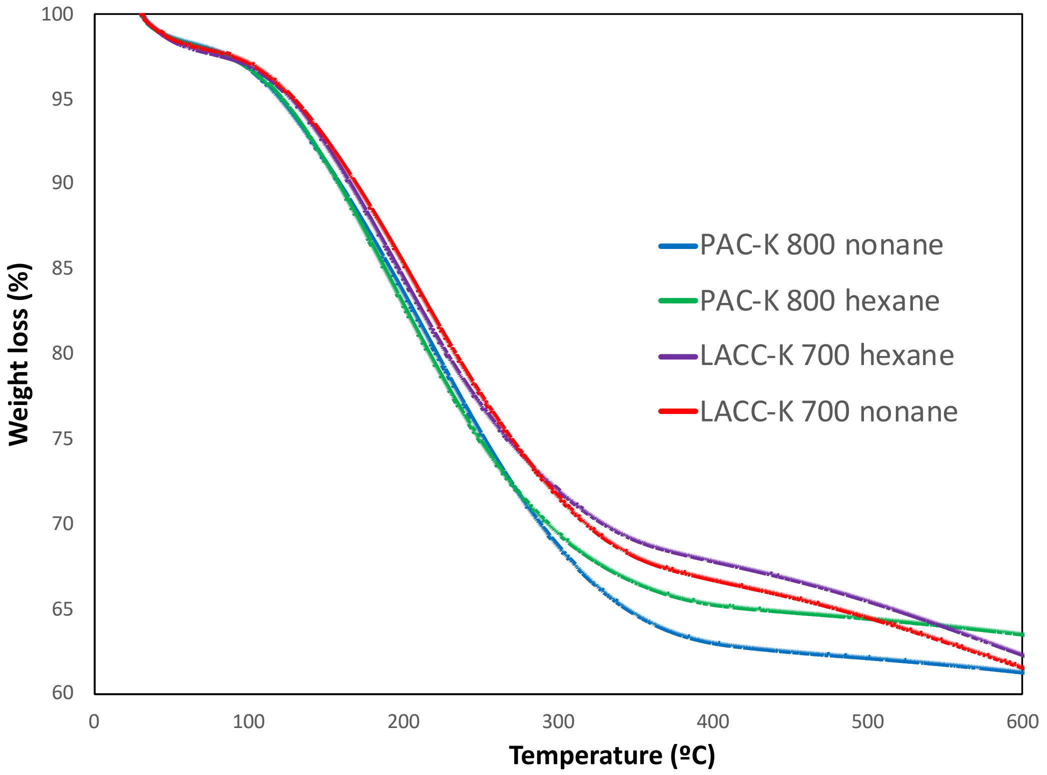

Figure S3. Thermogravimetric analysis (TG) of the LACC-K 700 and PAC-K 800 samples after pre-impregnation with nonane and hexane and a subsequent drying step at 60ºC for 1h under low vacuum.

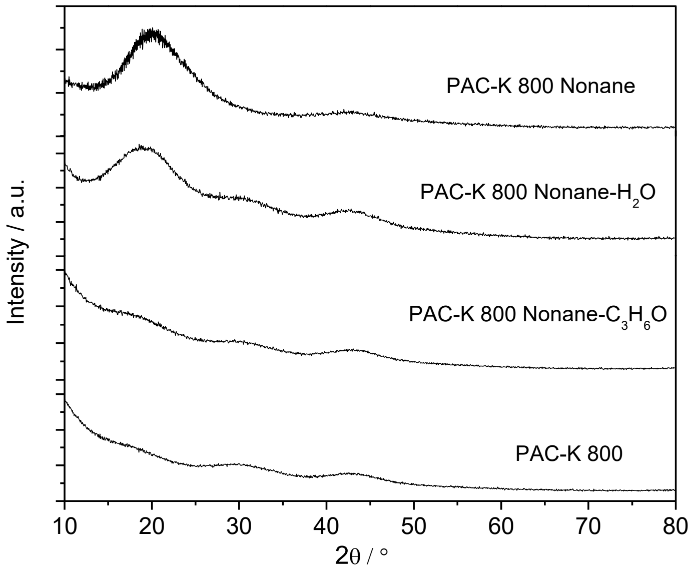

Figure S4. XRD pattern of the petroleum-based activated carbon (PAC-K 800) before and after n-nonane adsorption and after a washing treatment with H_2O and acetone (C_3H_6O).

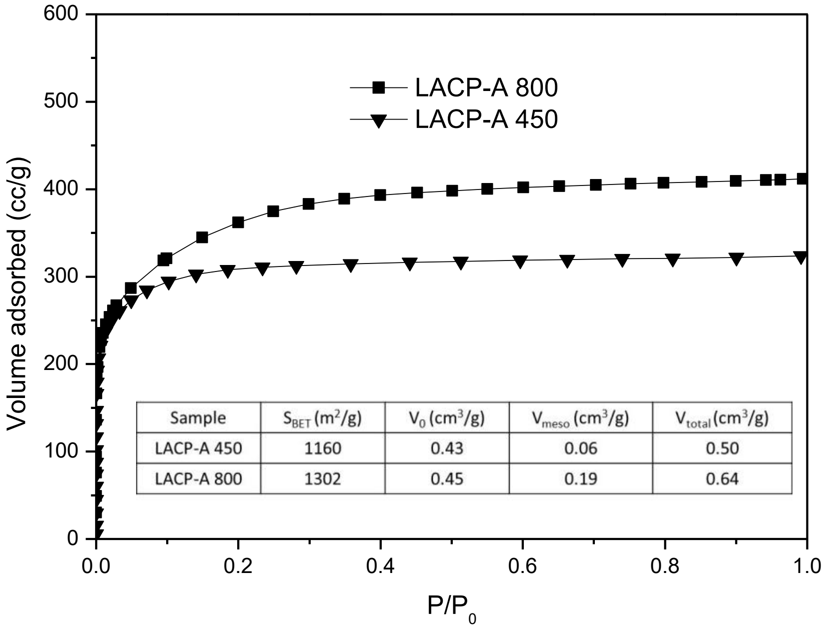

Sample	S_{BET} (m^2/g)	V_0 (cm^3/g)	V_{meso} (cm^3/g)	V_{total} (cm^3/g)
LACP-A 450	1160	0.43	0.06	0.50
LACP-A 800	1302	0.45	0.19	0.64

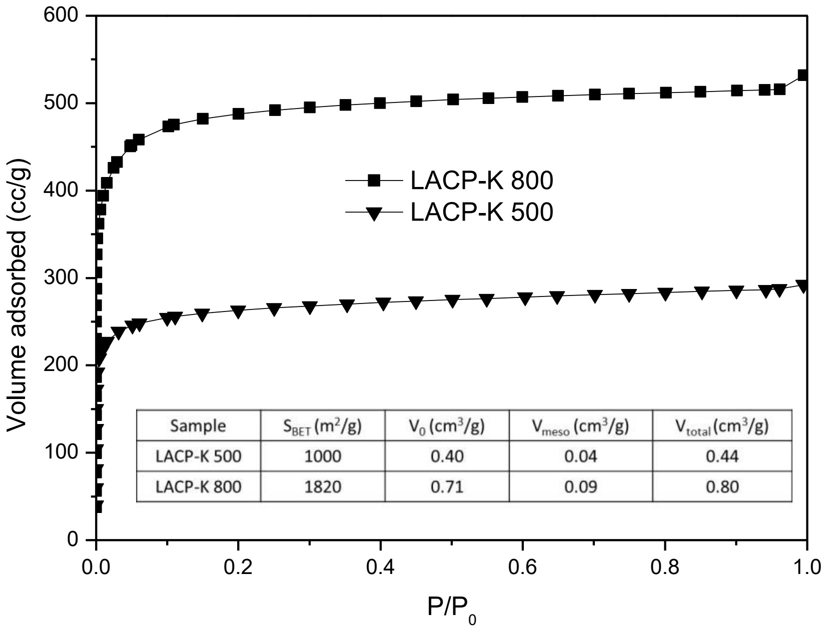

Figure S5. N_2 adsorption/desorption isotherms at 77 K for the peach stones derived activated carbons evaluated (LACP-A and LACP-K) prepared at low and high activation temperatures.

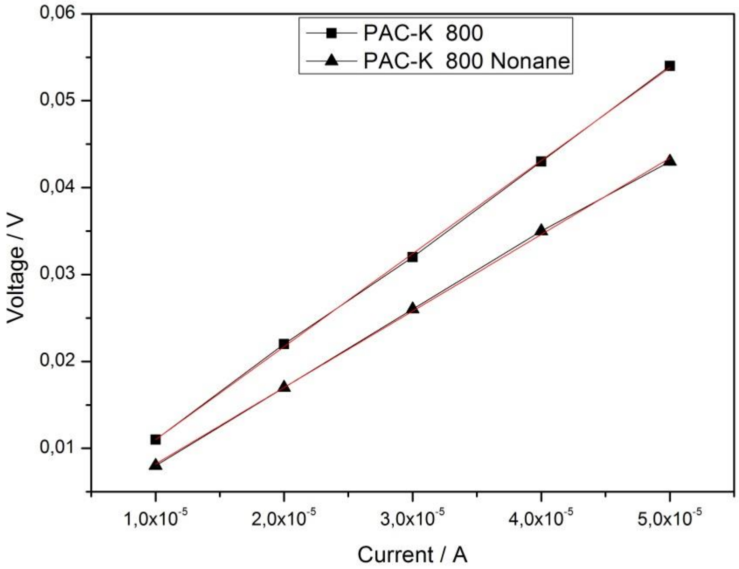

Figure S6. DC electrical resistivity measurements for PAC-K 800 sample before and after n-nonane adsorption using fourpoint probe method. The resistance values were obtained from the slope of the voltage versus current curves in ohmic regime.

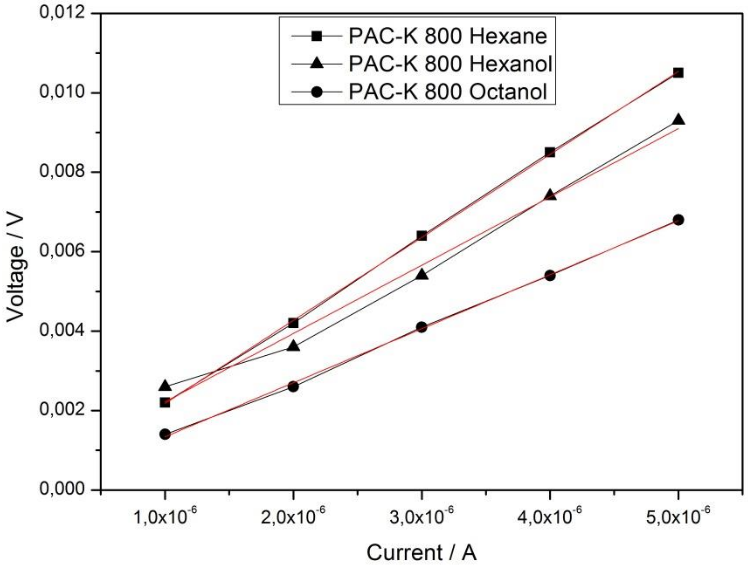

Figure S7. DC electrical resistivity measurements for PAC-K 800 sample after adsorption of hexane, hexanol and octanol using fourpoint probe method. The resistance values were obtained from the slope of the voltage versus current curves in ohmic regime.

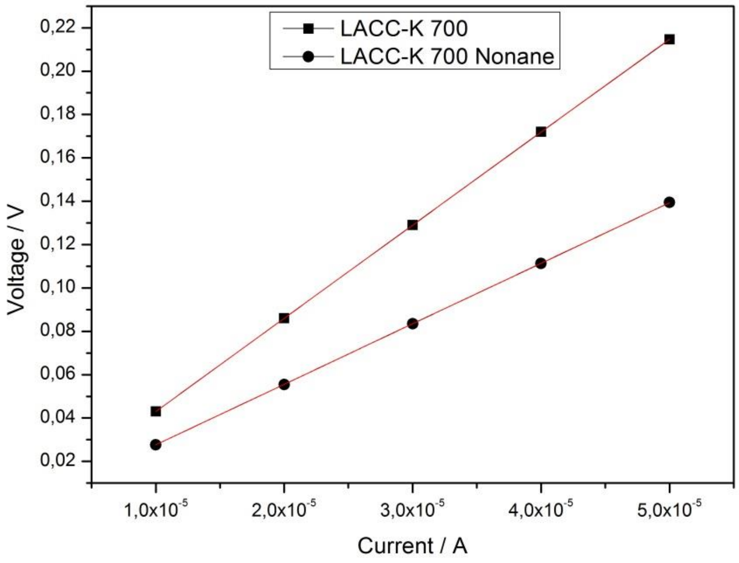

Figure S8. DC electrical resistivity measurements for LACC-K 700 sample before and after n-nonane adsorption using fourpoint probe method. The resistance values were obtained from the slope of the voltage versus current curves in ohmic regime.

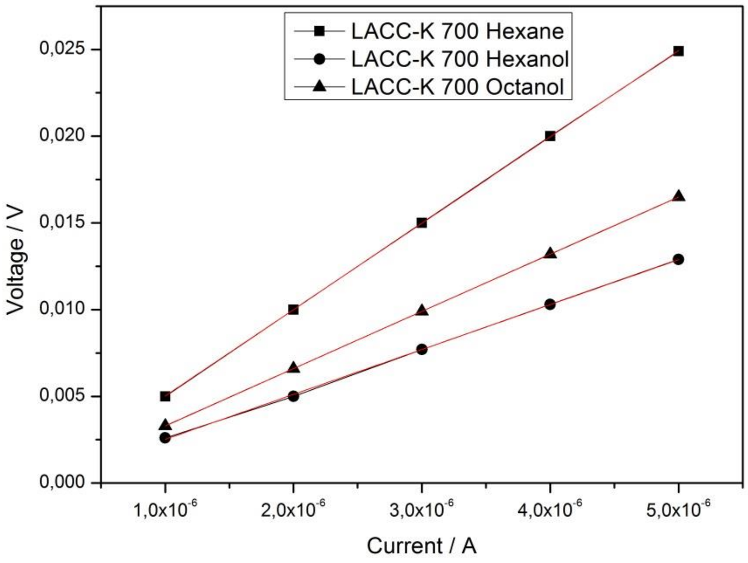

Figure S9. DC electrical resistivity measurements for LACC-K 700 sample after adsorption of hexane, hexanol and octanol using fourpoint probe method. The resistance values were obtained from the slope of the voltage versus current curves in ohmic regime.

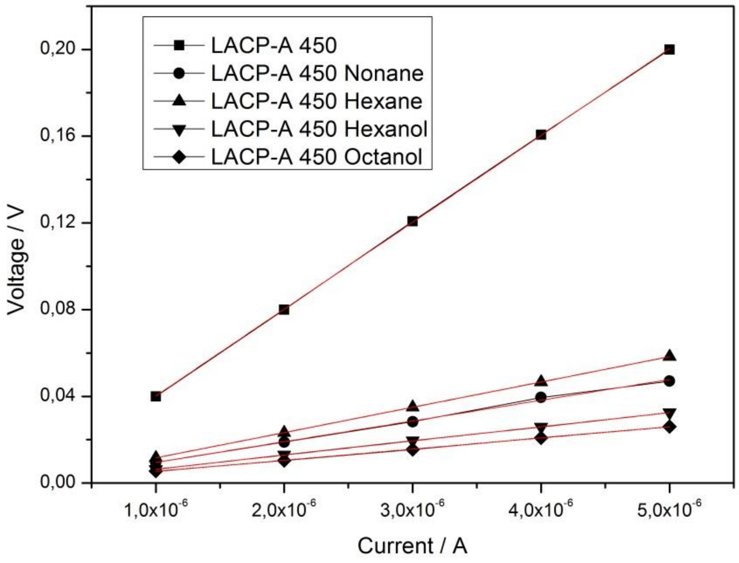

Figure S10. DC electrical resistivity measurements for LACP-A 450 before and after adsorption of n-nonane, hexane, hexanol and octanol using fourpoint probe method. The resistance values were obtained from the slope of the voltage versus current curves in ohmic regime.
